# Relationship between mandibular symphysis dimensions and mandibular anterior alveolar bone thickness as assessed with cone-beam computed tomography

**DOI:** 10.1590/2177-6709.23.1.054-062.oar

**Published:** 2018

**Authors:** Pimchanok Foosiri, Korapin Mahatumarat, Soontra Panmekiate

**Affiliations:** 1 Chulalongkorn University, Department of Orthodontics (Bangkok, Thailand).; 2 Chulalongkorn University, Department of Radiology (Bangkok, Thailand).

**Keywords:** Cone-beam computed tomography, Incisor, Chin, Mandible, Orthodontics

## Abstract

**Objective:**

To determine the relationship between symphysis dimensions and alveolar bone thickness (ABT) of the mandibular anterior teeth.

**Methods:**

Cone-beam computed tomography images of 51 patients were collected and measured. The buccal and lingual ABT of the mandibular anterior teeth was measured at 3 and 6 mm apical to the cemento-enamel junction (CEJ) and at the root apices. The symphysis height and width were measured. The symphysis ratio was the ratio of symphysis height to symphysis width. Kendall’s tau correlation coefficient was used to determine the relationships between the variables at a 0.05 significance level.

**Results:**

The mandibular anterior teeth lingual and apical ABT positively correlated with symphysis width (*p*<0.05). Moreover, these thicknesses negatively correlated with the symphysis ratio (*p*<0.05). Symphysis widths and ratios showed higher correlation coefficients with total and buccal apical ABT, compared with lingual ABT. Buccal ABT at 3 and 6 mm apical to the CEJ was not significantly correlated with most symphysis dimensions. The mean thickness of the buccal alveolar bone at the upper root half was only 0.2-0.6 mm, which was very thin, when compared with other regions.

**Conclusion:**

For mandibular anterior teeth, the apical alveolar bone and lingual alveolar bone tended to be thicker in patients with a wide and short symphysis, compared to those with a narrow and long symphysis. Buccal alveolar bone was, in general, very thin and did not show a significant relationship with most symphysis dimensions.

## RESUMO

**Objetivo:**

o objetivo desse artigo foi avaliar a correlação entre as dimensões da sínfise e a espessura do osso alveolar (EOA) na região anterior da mandíbula.

**Métodos:**

imagens de tomografia computadorizada de feixe cônico (TCFC) de 51 pacientes foram selecionadas e medidas. As EOAs vestibular e lingual dos dentes anteroinferiores foram medidas a 3 mm e a 6 mm para apical da junção cemento-esmalte (JCE) e nos ápices radiculares. A altura e a largura da sínfise foram medidas, e calculou-se a proporção entre ambas, chamada de proporção da sínfise. O coeficiente de correlação *tau* de Kendall foi utilizado para determinar a correlação entre as variáveis, com nível de significância de 0,05.

**Resultados:**

as EOAs vestibular e lingual dos dentes anteroinferiores apresentaram correlação positiva com a largura da sínfise (*p*< 0,05). Porém, essas EOAs apresentaram correlação negativa com a proporção da sínfise (*p* <0,05). A largura e a proporção da sínfise apresentaram maiores coeficientes de correlação com a EOA total e vestibular apical e, em comparação com a EOA lingual. A EOA vestibular a 3 mm e a 6 mm para apical da JCE não apresentou correlação significativa com a maioria das dimensões da sínfise. A espessura média do osso alveolar vestibular na metade superior da raiz foi de apenas 0,2 a 0,6 mm - muito delgada quando comparada com outras regiões.

**Conclusão:**

nos dentes anteroinferiores, o osso alveolar apical e o osso alveolar lingual tendem a ser mais espessos nos pacientes com sínfise mais larga e curta, em comparação àqueles com sínfise mais estreita e longa. O osso alveolar vestibular foi, em geral, muito fino e não apresentou relação significativa com a maioria das dimensões da sínfise.

## INTRODUCTION

Orthodontic tooth movement (OTM) occurs from the biological response of alveolar bone to pressure and tension, i.e., resorption and apposition, respectively. Studies on secondary remodeling and tooth movement found decreased alveolar bone thickness and root perforations of the lingual cortical plates when anterior teeth were moved in an anteroposterior direction.^1^ ^-^ ^3^ These results corresponded with those of Handelman,^4^ which indicated that iatrogenic sequelae, such as root perforation, dehiscence or fenestration, may occur due to teeth moving beyond the dimensions of the alveolus. Proffit et al^5^ proposed a theoretical model (“envelopes of discrepancy”) that suggested that orthodontic movement without surgery or growth modification produced the least tooth movement due to anatomical limitations.

To determine the therapeutic limits of OTM, several studies examined alveolar bone thickness (ABT). Both buccal and lingual bone tended to be very thin in the mandibular incisor region, especially at the upper root half.^6^ ^,^ ^7^ Additionally, bone dehiscence and fenestration prior to orthodontic treatment was commonly found in anterior regions, particularly in the mandibular incisor area, where thin alveolar bone support was seen.^8^ ^,^ ^9^ Consequently, ABT, especially in the mandibular incisor area should be taken into consideration to avoid iatrogenic complications and minimize periodontal tissue and tooth structure damage during orthodontic treatment. 

Prior studies demonstrated a relationship between vertical facial types and alveolar bone support at different tooth levels. Several studies concluded that long-face patients frequently showed thinner anterior alveolar bone at the root apex compared with normal-face and short-face patients^4^ ^,^ ^9^ ^-^ ^11^ Furthermore, a thin anterior alveolus was typical in normal-face Class III patients due to the dentoalveolar compensatory mechanism,^4^ ^,^ ^10^ ^,^ ^12^ and in patients with severe bimaxillary protrusion.^4^ Although thin apical alveolar bone was more frequently found in long lower facial height patients, it could be encountered in any other skeletal types.^4^

Bone thickness measurements in most previous studies were limited to the root apex level.^4^ ^,^ ^7^ ^,^ ^9^ ^-^ ^11^ Sarikaya et al^1^ stated that both buccal and lingual marginal alveolar bone loss was inevitable during mandibular anterior teeth retraction. Accordingly, marginal and mid-root alveolar bone widths are as important as apical widths and should be taken into consideration when planning orthodontic treatment.^1^ Hoang et al^13^ concluded that the difference in bucco-lingual bone thickness at the alveolar crest was less pronounced than that at the root apex among the three vertical skeletal patterns.^13^ Additionally, buccal and lingual ABT at the cervical and middle thirds of the root was similar for both hyperdivergent and hypodivergent vertical facial patterns.^14^ Similarly, both buccal and lingual ABT at the middle root third demonstrated a weak correlation with vertical facial patterns.^15^ Importantly, thin anterior alveolus could be found in any skeletal types.^4^ Consequently, there may be other factors related to mandibular anterior bone support, especially in the upper root half, apart from vertical facial types. Wehrbein et al^16^ showed that symphysis morphology might relate to alveolar bone support of the mandibular anterior teeth. Progressive alveolar support loss was found in an orthodontic patient with a narrow and long symphysis. However, the association between symphysis morphology and mandibular anterior alveolar bone support remains unsolved. 

Lateral cephalometric radiography (LCR) has long been used to examine alveolar bone thickness. However, three-dimensional structures overlap in 2D images. Furthermore, 2D radiographs produce a magnification error due to X-ray beam divergence.^17^ Thus, assessing mandibular ABT from LCR is unreliable due to overlapping in the incisor region. Cone-beam computed tomography (CBCT) provides three-dimensional data with higher accuracy and reliability, allowing for dimensional measurements that correspond to actual anatomical measurements.^18^ ^,^ ^19^ This technique could be useful in assessing quantitative and qualitative alveolar bone morphology data.^19^

Currently, there are no reports using CBCT data to evaluate the correlation between mandibular symphysis dimensions and mandibular anterior ABT at various tooth levels, including coronal and mid-root. The aim of this study was to evaluate the relationship between symphysis dimensions and ABT of the mandibular anterior teeth using CBCT at the cervical, middle and apical root thirds, in a broad sample of patients.

## MATERIALS AND METHODS

From 1,988 patients, whose CBCT images were acquired from January 2014 to March 2016, 51 consecutive subjects (21 males, mean age 26.19 years; 30 females, mean age 25.44 years) meeting the inclusion criteria were collected, resulting in a sample size of 306 mandibular anterior teeth. The inclusion criteria were subjects aged 18-35 years old, CBCT images displaying the entire mandibular symphysis and all mandibular anterior teeth regions, regardless of vertical skeletal pattern and type of occlusion. Subjects with prior orthodontic treatment, >3 mm of mandibular anterior crowding or blocked out teeth, periodontal disease, missing lower anterior teeth, or pathology that might affect the mandible and alveolar bone, were excluded. The data of the mandibular teeth were collected and separated into the following groups, divided into left and right sides: central incisors, lateral incisors, and canines. The CBCT images were acquired using 3D Accuitomo 170 machine (J. Morita, Kyoto, Japan) using 90 kV, 5 mA, 17.5 s exposure time, and a field of view of 8 x 8 or 10 x 10 cm, resulting in voxel sizes of 0.165 and 0.25 mm, respectively. Each CBCT scan was taken as part of treatment and diagnosis, including implant-site assessment and embedded tooth localization; therefore, no subjects received an unjustified radiation exposure. The study protocol was approved by the University Ethics Committee (HREC-DCU 2015-096). 

**Figure 1:**
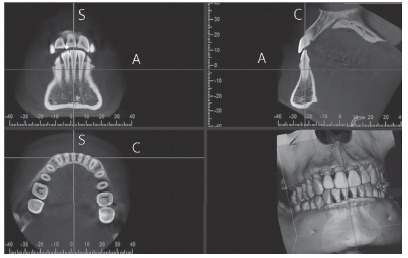
Lower anterior tooth sagittal cross-section construction using I-Dixel Software. The sagittal slice was positioned through the long axis of each lower anterior tooth, perpendicular to the curvature of the alveolar ridge. The sagittal cross-section (upper right image) was used to measure alveolar bone thickness. A, C, and S represent the lines corresponding to the axial, coronal, and sagittal planes, respectively.

**Figure 2:**
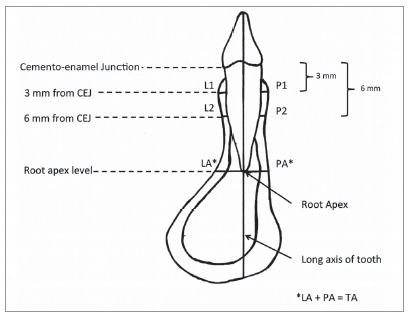
Sagittal cross-section of the lower anterior tooth.

**Figure 3:**
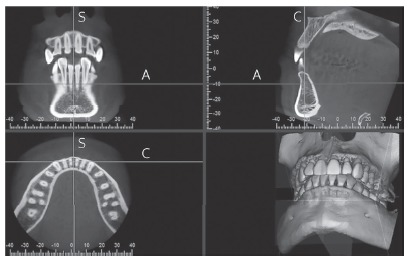
Mandibular symphysis sagittal cross-section construction using I-Dixel software. The sagittal slice was positioned through the mandibular midline. The sagittal cross-section (upper right image) was used for symphysis dimensions measurements. A, C, and S represent the lines corresponding to the axial, coronal, and sagittal planes, respectively.

**Figure 4:**
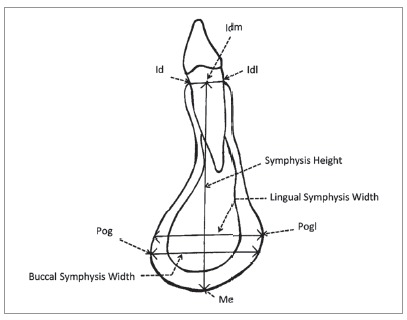
Sagittal cross-section of the mandibular symphysis displaying symphysis region landmarks and variables.

I-Dixel One Volume Viewer Software (V. 2.0.0, J. Morita) was used for viewing and measuring images by a single operator who had been trained, and under the supervision of a certified oral and maxillofacial radiologist. A 1-mm slice thickness was used. For bone thickness measurements, the sagittal slice was positioned through the long axis of each tooth, perpendicular to the alveolar ridge curvature (Fig 1). Buccal and lingual ABT of the mandibular anterior teeth was measured from the root surface to the external limit of the mandibular buccal and lingual cortex, perpendicular to the long axis of each tooth, 3 and 6 mm apical to the cemento-enamel junction (CEJ) and at the root apices (Fig 2). FDI tooth numbering system was used to identify each tooth. For the symphysis dimension measurements, a sagittal slice was placed along the mandibular midline (Fig 3). The symphysis height was measured from midpoint of anterior alveolus (Idm) to Menton (Me). The buccal symphysis width was measured from the buccal pogonion (Pog) to the external limit of the lingual cortex, perpendicular to the symphysis height. The lingual symphysis width was measured from the lingual pogonion (Pogl) to the external limit of the buccal cortex, perpendicular to the symphysis height (Fig 4, Table 1). The buccal symphysis ratio was calculated by dividing the symphysis height by the buccal symphysis width. The lingual symphysis ratio was calculated by dividing symphysis height by the lingual symphysis width. One month after the first measurement, 20% of the subjects were selected at random and all variables were measured again. An intraclass correlation coefficient of 0.91-0.99 was found, showing excellent intra-rater reliability.

Bone thickness was measured perpendicular to the long axis of the tooth. 

Variables: L1, buccal bone thickness 3 mm apical to the CEJ; L2, buccal bone thickness 6 mm apical to the CEJ; LA, buccal bone thickness at the root apex; P1, lingual bone thickness 3 mm apical to the CEJ; P2, lingual bone thickness 6 mm apical to the CEJ; PA, lingual bone thickness at the root apex; TA, total apical bone thickness, LA+PA, sum of the buccal and lingual bone thickness at the root apex.

**Table 1:** Landmarks and variables of symphysis region.

Abbreviation	Name	Definition
Id	Infradentale	The most superior anterior point on mandibular alveolar process between central incisors
Idl	Lingual point of infradentale	The most superior posterior point on mandibular alveolar process of tooth between central incisors
Me	Menton	The most inferior point of mandibular symphysis
Pog	Buccal Pogonion	The most anterior point of mandibular symphysis
Pogl	Lingual Pogonion	The most convex point of lingual curvature of symphysis
Idm*	Midpoint of anterior alveolus	Midpoint of line drawn from Id to Idl
-	Buccal symphysis width	Total width of mandibular symphysis measured from buccal pogonion to the external limit of lingual cortex perpendicular to symphysis height
-	Lingual symphysis width	Total width of mandibular symphysis measured from lingual pogonion to the external limit of buccal cortex perpendicular to symphysis height
-	Symphysis height	Linear distance from Idm to Me
-	Buccal symphysis ratio	Ratio of symphysis height to buccal symphysis width
-	Lingual symphysis ratio	Ratio of symphysis height to lingual symphysis width

*Based on Suri et al.^20^

### Statistical analysis

The Mann-Whitney U test was used to analyze the difference between the male and female subjects’ variables. The variables of the same tooth were compared between the right and left sides by the Wilcoxon Signed-Rank test. The Kolmogorov-Smirnoff test was used to determine the normality of the data, which were not normally distributed. Therefore, Kendall’s tau correlation coefficient was used to determine the relationship between the symphysis dimensions and ABT of the mandibular anterior teeth. A*p*< 0.05 was considered significant for all tests. The statistical analyses were performed with SPSS software package (IBM SPSS Statistics for Windows, version 22.0. Armonk, NY: IBM Corp.).

## RESULTS

No significant difference was found between the male and female variables; therefore, the data were combined for subsequent analysis. The ABT measurements between the left and right sides were not significantly different, with the exception of the following: (1) lingual alveolar bone at the mandibular central incisor root apex (PAx31 and PAx41), (2) lingual alveolar bone 6 mm from the CEJ of the mandibular lateral incisors (P2x32 and P2x42), (3) lingual alveolar bone 3 mm from the CEJ of the mandibular canines (P1x33 and P1x43). Consequently, the measurements of these three pairs were analyzed separately as left and right values. The other pairs were combined (Table 2). Symphysis dimensions of the subjects are illustrated in Table 3.

**Table 2:** Mean alveolar bone thickness for lower anterior teeth

Variables*	Mean	Std. Deviation
L1xCentral	0.56	0.27
L2xCentral	0.36	0.17
LAxCentral	3.63	1.22
P1xCentral	0.38	0.22
P2xCentral	0.80	0.55
PAx31	4.44	1.27
PAx41	4.24	1.14
TAxCentral	7.97	1.91
L1xLateral	0.58	0.33
L2xLateral	0.27	0.15
LAxLateral	3.99	1.39
P1xLateral	0.49	0.30
P2x32	1.28	0.83
P2x42	1.08	0.70
PAxLateral	4.39	1.18
TAxLateral	8.38	2.02
L1xCanine	0.40	0.23
L2xCanine	0.25	0.10
LAxCanine	4.49	1.56
P1x33	1.33	0.90
P1x43	1.09	0.68
P2xCanine	2.25	1.06
PAxCanine	5.53	1.44
TAxCanine	10.02	2.00

*L1, L2, LA, P1, P2, PA, TA: see these sites in Figure 2.

*Central, Lateral, Canine: means mandibular central incisors, lateral incisors and canines, respectively.

*31, 32, 33, 41, 42, 43: refer to the teeth according to the FDI tooth numbering system.

**Table 3:** Mean and standard deviation of symphysis dimensions.

Variables	Mean	Std. Deviation
Buccal symphysis width	13.54	1.71
Lingual symphysis width	14.24	1.88
Symphysis height	32.13	2.55
Buccal symphysis ratio	2.41	0.33
Lingual symphysis ratio	2.29	0.34

### Symphysis width and height

Buccal symphysis width showed a positive correlation with the buccal, lingual and total ABT at the root apices of all mandibular anterior teeth. Buccal symphysis width also positively correlated with lingual ABT 6 mm apical to the CEJ of all teeth, and lingual ABT 3 mm apical to the CEJ for canines (P1xCanine). Lingual symphysis width demonstrated a similar relationship, with a weaker correlation compared with the buccal symphysis width, except for the lingual ABT 3 mm apical to CEJ for the lower right canine (P1x43). In contrast, the symphysis height was not significantly correlated with most ABT measurements. No significant relationship was found between most symphysis dimensions and buccal ABT 3 mm or 6 mm apical to the CEJ (Tables 4, 5 and 6).

**Table 4:** Correlation between buccal symphysis/ lingual symphysis and mandibular central incisor alveolar bone thickness.

		L1xCentral	L2xCentral	LAxCentral	P1xCentral	P2xCentral	PAx31	PAx41	TAxCentral
Buccal symphysis width	Correlation Coefficient	0.028	0.03	0.365**	0.094	0.298**	0.352**	0.352**	0.475**
	Sig. (2-tailed)	0.776	0.763	0.000	0.333	0.002	0.000	0.000	0.000
Lingual symphysis width	Correlation Coefficient	0.090	0.123	0.234*	0.157	0.263**	0.291**	0.270**	0.339**
	Sig. (2-tailed)	0.358	0.212	0.016	0.107	0.007	0.003	0.006	0.000
Symphysis height	Correlation Coefficient	0.133	0.203*	-0.227*	-0.088	-0.133	0.057	0.052	-0.075
	Sig. (2-tailed)	0.174	0.039	0.019	0.367	0.170	0.558	0.592	0.440
Buccal symphysis ratio	Correlation Coefficient	0.037	0.058	-0.478**	-0.207*	-0.360**	-.303**	-0.296**	-0.501**
	Sig. (2-tailed)	0.708	0.557	0.000	0.033	0.000	0.002	0.002	0.000
Lingual symphysis ratio	Correlation Coefficient	-0.002	-0.035	-0.390**	-0.220*	-0.331**	-0.232*	-0.217*	-0.405**
	Sig. (2-tailed)	0.987	0.720	0.000	0.024	0.001	0.017	0.025	0.000

** Correlation is significant at the 0.01 level (2-tailed).

* Correlation is significant at the 0.05 level (2-tailed).

See Table 2 legend for abbreviation explanation.

**Table 5:** Correlation between buccal symphysis/ lingual symphysis and mandibular lateral incisor alveolar bone thickness.

		L1xLateral	L2xLateral	LAxLateral	P1xLateral	P2x32	P2x42	PAxLateral	TAxLateral
Buccal symphysis width	Correlation Coefficient	0.056	0.027	0.361**	0.150	0.298**	0.216*	0.383**	0.518**
	Sig. (2-tailed)	0.564	0.788	0.000	0.124	0.002	0.027	0.000	0.000
Lingual symphysis width	Correlation Coefficient	0.086	0.103	0.225*	0.155	0.263**	0.193*	0.322**	0.377**
	Sig. (2-tailed)	0.380	0.297	0.021	0.113	0.007	0.049	0.001	0.000
Symphysis height	Correlation Coefficient	0.146	0.098	-0.322**	-0.178	-0.097	-0.066	0.214*	-0.074
	Sig. (2-tailed)	0.135	0.320	0.001	0.067	0.317	0.500	0.027	0.445
Buccal symphysis ratio	Correlation Coefficient	0.029	0.030	-0.509**	-0.264**	-0.330**	-0.247*	-0.238*	-0.522**
	Sig. (2-tailed)	0.764	0.763	0.000	0.007	0.001	0.011	0.014	0.000
Lingual symphysis ratio	Correlation Coefficient	0.012	-0.039	-0.413**	-0.292**	-0.317**	-0.251*	-0.194*	-0.431**
	Sig. (2-tailed)	0.903	0.690	0.000	0.003	0.001	0.010	0.045	0.000

** Correlation is significant at the 0.01 level (2-tailed).

* Correlation is significant at the 0.05 level (2-tailed).

See Table 2 legend for abbreviation explanation.

### Symphysis ratio

The buccal and lingual symphysis ratios (ratio of height/width) negatively correlated with the buccal, lingual and total ABT at the root apices for almost all teeth, except for the lingual ABT at the canine root apices (PAxCanine). Both ratios also negatively correlated with lingual ABT 3 and 6 mm apical to the CEJ for all teeth. Buccal symphysis ratio mostly showed a higher correlation compared with the lingual symphysis ratio. There was no significant relationship between buccal or lingual symphysis ratios and buccal ABT 3 mm or 6 mm apical to the CEJ (Tables 4, 5 and 6).

**Table 6:** Correlation between buccal symphysis/ lingual symphysis and mandibular canine alveolar bone thickness.

		L1xCanine	L2xCanine	LAxCanine	P1x33	P1x43	P2xCanine	PAxCanine	TAxCanine
Buccal symphysis width	Correlation Coefficient	0.086	0.140	0.380**	0.280**	0.212*	0.307**	0.264**	0.497**
	Sig. (2-tailed)	0.375	0.158	0.000	0.004	0.030	0.002	0.006	0.000
Lingual symphysis width	Correlation Coefficient	0.128	0.261**	0.285**	0.215*	0.162	0.235*	0.269**	0.424**
	Sig. (2-tailed)	0.190	0.009	0.003	0.028	0.099	0.016	0.006	0.000
Symphysis height	Correlation Coefficient	0.067	0.059	-0.214*	-0.113	-0.070	0.006	0.235*	-0.025
	Sig. (2-tailed)	0.490	0.552	0.027	0.248	0.474	0.955	0.015	0.795
Buccal symphysis ratio	Correlation Coefficient	-0.087	-0.079	-0.480**	-0.339**	-0.263**	-0.272**	-0.126	-0.453**
	Sig. (2-tailed)	0.371	0.424	0.000	0.001	0.007	0.005	0.194	0.000
Lingual symphysis ratio	Correlation Coefficient	-0.095	-0.203*	-0.395**	-0.310**	-0.233*	-0.247*	-0.135	-0.400**
	Sig. (2-tailed)	0.329	0.040	0.000	0.001	0.017	0.011	0.162	0.000

** Correlation is significant at the 0.01 level (2-tailed).

* Correlation is significant at the 0.05 level (2-tailed).

See Table 2 legend for abbreviation explanation.

## DISCUSSION

The results of the present study demonstrated a positive correlation between symphysis widths and apical ABT, as well as lingual ABT at the middle root third. Moreover, apical ABT and lingual ABT at the cervical and middle thirds of the roots negatively correlated with symphysis ratios. The wider the symphysis, the thicker the apical and lingual alveolar bone tended to be. The smaller the symphysis ratio, which represents a short and wide symphysis, the thicker the apical and lingual alveolar bone tended to be. These findings partially conformed to a study demonstrating that mandible with a long and narrow symphysis underwent progressive loss of both buccal and lingual bone due to thinner alveolar bone support.^16^

Lingual symphysis width and ratio showed a weaker correlation with ABT, compared with their buccal counterparts. The buccal symphysis ratio significantly correlated with the lingual ABT 3 and 6 mm apical to the CEJ for all teeth, while the buccal symphysis width showed a significant relationship with the lingual ABT 6 mm apical to the CEJ for all teeth and lingual ABT 3 mm apical to the CEJ for canines only. Consequently, the parameters that showed the strongest relationships with ABT were the buccal symphysis ratio and buccal symphysis width, respectively.

In the present study, the buccal symphysis ratio tended to have a stronger relationship with the buccal and total ABT at root apices, compared with the lingual ABT at every level. The significant correlation coefficients between the buccal symphysis ratio and lingual ABT at 3 mm and 6 mm apical to the CEJ, and at the root apices ranged from 0.207 to 0.360. The correlation coefficients between the buccal symphysis ratio and total apical ABT, as well as buccal apical ABT, ranged from 0.453 to 0.522 and 0.478 to 0.509, respectively. This suggests that patients with a wide and short symphysis tended to have thicker apical and lingual alveolar bone than those with a narrow and long symphysis. Evaluating symphysis morphology before initiating orthodontic treatment might help orthodontists to estimate the mandibular anterior teeth bony support and design an appropriate treatment plan. Patients with a wide and short symphysis might allow more lingual tooth movement within the anatomical limits than those with a narrow and long symphysis. The possibility of estimating total and buccal apical ABT might be stronger than for lingual ABT, because the former presented a stronger relationship with the buccal symphysis ratio. However, the present results showed only a tendency for the correlations. Orthodontists should keep in mind that the correlation coefficients between the symphysis dimensions and ABT in this study were not high enough to accurately predict alveolar bone support based only on symphysis dimensions. 

No significant relationships were found between most buccal ABT at 3 and 6 mm apical to the CEJ and symphysis dimensions. However, mean buccal ABT at 3 and 6 mm apical to CEJ tended to be thin (0.4-0.6 and 0.2-0.4 mm, respectively). These results corresponded with those of several studies that documented thin buccal alveolar bone at the mandibular anterior region, especially at the upper root half.^6^ ^-^ ^8^ Similarly, dehiscence was also found, primarily at the cervical third of the buccal alveolar bone of the mandibular anterior region.^21^ The majority of fenestrations were observed at the upper part of the buccal bone plates of mandibular incisors.^22^ Therefore, orthodontic buccal movement of the mandibular anterior teeth should be performed with great care, irrespective of symphysis dimensions.

According to a study of postnatal mandibular growth patterns, the mental protuberance of the chin, together with the lingual cortex of the anterior mandible, showed accumulative periosteal bone deposition.^23^ The buccal cortex superior to the mental protuberance exhibited variable degrees of periosteal bone resorption, ranging from restricted resorption at the interdental area to an entirely resorbed periosteal surface. This study showed comparable bone remodelling activity between the anterior mandibular lingual cortical bone and the mental protuberance. This might explain the positive association we found between the lingual ABT and the symphysis width. The fact that the buccal ABT at the upper root half did not show a significant relationship with most symphysis dimensions might be due to the differences in bone remodelling between these areas and a variable degree of periosteal bone resorption at the buccal cortex superior to the mental protuberance.

Some studies investigated symphysis width by measuring ABT at the root apices of the mandibular central incisors.^7^ ^,^ ^10^ ^,^ ^13^ The measurements at the root apex level generally presented smaller widths, compared with the measurements at the mental protuberance, and were influenced by the variation in mandibular incisor root length. A prior study demonstrated that mandibular central incisor root length ranged from 9.13 to 17.24 mm.^24^ In the present study, symphysis width was measured at the pogonion level, while the ABT at the root apices was defined as total apical ABT. Prior studies determined average symphysis width at the pogonion using CBCT and LCR. Beaty and Le^25^ demonstrated mean symphysis width using CT images of the head and neck region of 14.03±1.53 mm and 13.21±1.46 mm for men and women, respectively. Another study found that the mean symphysis width of Caucasian Brazilian adults with a well-balanced face and normal occlusion measured from LCR was 15.61 mm, with no significant difference between sexes.^26^ Compared with the present findings, the wider symphysis thickness measured in that study might result from LCR image magnification, different ethnic origin, and measuring methodology. They measured the distance from the buccal to the lingual pogonion, whereas the buccal symphysis width in the present study was derived from the perpendicular distance from the buccal pogonion to its counterpart, which might not be the most posterior point of the lingual curvature. 

Numerous studies have investigated the relationships between vertical facial patterns and mandibular anterior ABT at the root apices.^4^ ^,^ ^7^ ^,^ ^9^ ^-^ ^11^ ^,^ ^13^ Some studies showed that ABT, particularly in the upper root half, might not related to vertical facial patterns^13^ ^-^ ^15^, and thin anterior alveolus could be found in other skeletal types, apart from hyperdivergent faces.^4^ Thus, the present study evaluated other factors that might correlated with ABT at various tooth levels. The main objective was to examine whether the relationships existed between symphysis dimensions and mandibular anterior ABT, which had not yet been reported. It was found a significant correlation between certain symphysis dimensions and ABT at the cervical, middle and apical root thirds, in a broad sample of consecutive subjects. To expand the understanding of these relationships, further studies with larger sample sizes are indicated to investigate the correlations in subjects with different skeletal patterns.

## CONCLUSION

The symphysis widths of the mandibular anterior teeth positively correlated with total, buccal and lingual ABT at the root apices and lingual ABT at the middle root third. Symphysis ratios, which are ratios of symphysis height to symphysis width, negatively correlated with total, buccal and lingual ABT at the root apices and lingual ABT at the cervical and middle root thirds. Therefore, apical alveolar bone and lingual alveolar bone tended to be thicker in patients with a wide and short symphysis compared with those with a narrow and long symphysis. Buccal alveolar bone at the cervical and middle thirds of the roots was, in general, thin and showed no significant correlation with most symphysis dimensions.
